# Targeting the P2X7 receptor in cerebrovascular diseases: from molecular mechanisms to preclinical therapeutic potential

**DOI:** 10.3389/fphar.2025.1713748

**Published:** 2025-11-26

**Authors:** Ziyan Hu, Shujing Xie, Cheng Chen, Zhihui Luo, Xiao Deng, Renjie Xiao

**Affiliations:** 1 Department of Anesthesiology, The Second Affiliated Hospital of Nanchang University, Nanchang, Jiangxi, China; 2 The Second Clinical Medical College of Nanchang University, Nanchang, Jiangxi, China; 3 Nanchang University Queen Mary School, Nanchang, Jiangxi, China; 4 Department of Operating Room, The Second Affiliated Hospital of Nanchang University, Nanchang, Jiangxi, China

**Keywords:** P2X7R, cerebrovascular diseases, pathogenesis, antagonists, pharmacology

## Abstract

Cerebrovascular diseases seriously damage human health and impose a huge burden on society. Research on the mechanisms of cerebrovascular disease occurrence and development is of great significance in preventing the occurrence of the disease and improving the quality of life of patients. P2X7 Receptor (P2X7R), as a non-selective cation channel of the purinergic receptor family, is considered to be expressed in various immune cells within the nervous system and may also be expressed in neurons. Recent studies have identified P2X7R as a significant player in the progression of cerebrovascular diseases, potentially linked to its role in regulating neuroinflammation, cellular autophagy, and vascular function. This review elucidates the biological foundation of P2X7R, compiles various molecular mechanisms associated with cerebrovascular diseases, emphasizes recent research on the involvement of P2X7R in the pathogenesis of cerebrovascular diseases, and assesses the pharmacological implications of P2X7R in these conditions. By exploring the connections between P2X7R and cerebrovascular diseases, the therapeutic potential of targeting P2X7R in these conditions can be assessed, ultimately paving the way for novel therapeutic strategies to ameliorate the impact of cerebrovascular diseases.

## 1 Introduction

Cerebrovascular diseases are diseases related to cerebral blood flow, mainly coronary artery disease (CAD), subarachnoid hemorrhage (SAH), intracerebral hemorrhage (ICH), stroke, and atherosclerosis (Zhao et al., 2016; Bai et al., 2021). Seizures of cerebrovascular diseases are often accompanied by complications, such as epilepsy as a complication of SAH (Wang J. et al., 2021). Most patients with stroke suffer from motor deficits, and a small number of patients with stroke develop cognitive deficits (Huang et al., 2017). A World Health Organization report shows that the second leading cause of death globally in 2019 is stroke (Ri et al., 2023). And the Global Burden of Disease Report 2023 shows that up to 7.2 million people lose their lives to CAD each year, affecting the normal lives of 126 million people (Figtree et al., 2023). Cerebrovascular diseases seriously jeopardize human life and health, and most patients’ families find it difficult to bear the high cost of treatment, and the patients suffer greatly from the disease. Therefore, effective prevention and treatment of cerebrovascular diseases are of great significance and social value to society, patients, and their families.

P2X7R is a class of ligand-gated cation channels that can be activated by adenosine triphosphate (ATP), allowing macromolecules to enter the cell inwardly. It can mediate Ca^2+^ inward flow and promote the maturation of the inflammatory factors interleukin (IL)-1β and IL-18, thereby promoting neuroinflammation and excitotoxicity (Wang J. et al., 2021; Ahn et al., 2023; Peng et al., 2015). P2X7R is selectively expressed in cerebral neurons and glial cells. Recent single-cell sequencing studies have shown that P2X7R is predominantly expressed in microglia, with very low expression in neurons (Beltran-Lobo et al., 2023a).

Existing studies have found that P2X7R has a close relationship with cerebrovascular diseases. For example, dextromethorphan (Ischemic Brain Injury Disease Drug, Dexmedetomidine) can downregulate the level of P2X7R and inhibit P2X7R to promote the process of microglial cell pyroptosis to achieve protection against ischemic brain injury (Sun et al., 2021). In another study, it was found that the reduction of neurological deficits, brain edema, and maintenance of blood-brain barrier integrity can be accomplished by inhibiting or knocking down P2X7R, but these potent effects are reversed when there is pretreatment with 2′(3′)O-(4-benzoylbenzoyl)-ATP (BzATP) (Zhao et al., 2016). Moreover, recent clinical trials suggest that P2X7R antagonists show potential efficacy in patients with ischemic stroke (Cisneros-Mejorado et al., 2015a). Therefore, targeting P2X7R presents a promising strategy for combating cerebrovascular diseases.

This review aims to elucidate the relevance of P2X7R to cerebrovascular disease and to explore possible methods for preventing and controlling these diseases. Meanwhile, a better understanding of the mechanism of P2X7R in cerebrovascular disease will facilitate the exploration of new therapeutic approaches and strategies to mitigate the impact of these diseases on human health.

## 2 Definition, epidemiology and common mechanisms of cerebrovascular disease

Cerebrovascular disease refers to brain tissue damage caused by impaired cerebral blood flow, which in turn results in clinical symptoms such as transient ischemic attack, stroke, and vascular dementia. Cerebrovascular diseases are commonly known as ischemic stroke and hemorrhagic stroke, which shows that stroke is the most common clinical manifestation of cerebrovascular diseases. Stroke includes cerebral hemorrhage, ischemic stroke, and subarachnoid hemorrhage. The World Stroke Organization’s Global Stroke Fact Sheet 2025 states that stroke is the second leading cause of death in the world and the third leading cause of death and disability (Feigin et al., 2025). The Lancet Neurology Commission predicts that between 2020 and 2050, an additional 3.1 million people will die from stroke, and the global economic cost of stroke is estimated at more than 891 billion dollars per year (Feigin et al., 2023).

In view of the great threat to human life and health, cerebrovascular diseases have been studied extensively for a long time around the world. The molecular mechanisms underlying the development of cerebrovascular diseases are numerous and complex. Exploration of the mechanisms of cerebrovascular disease development will be beneficial to the prevention, treatment and prognosis of the disease in the future. Currently, many studies have demonstrated that the common mechanisms of cerebrovascular disease include neuroinflammatory responses, oxidative stress, and cellular autophagy, which are involved in and contribute to neuronal death, blood-brain barrier disruption, cerebral vasospasm, and other pathological changes that lead to adverse outcomes in cerebrovascular disease. Inflammatory responses participate in the pathologic processes of many diseases, and inflammatory processes are often emphasized in the pathogenesis of cerebrovascular diseases. In studies of the relationship between various inflammatory factors and the prognosis of SAH, researchers have found that inhibition of the inflammatory response reduces neuronal apoptosis, cerebral vasospasm, and disruption brought about by the blood-brain barrier, which in turn attenuates brain injury (Hong et al., 2023; Shao et al., 2016; Yin et al., 2018). Likewise, in the study of ICH, it has been found that inflammatory processes lead to the onset of a number of pathophysiological processes such as neuronal death and disruption of the blood-brain barrier, leading to a poor prognosis in ICH (Fang et al., 2024). Oxidative stress, as another important mechanism, also plays an important role in cerebrovascular diseases. Among them, excessive ROS (reactive oxygen species) production is considered to be the root cause of oxidative stress. In both SAH and ICH, damage to proteins and nucleic acids by excess ROS was observed and ultimately led to cell death or apoptosis (Zhang Z. et al., 2022; Wan et al., 2019). Inhibition of oxidative stress, in turn, reduces apoptosis after cerebral ischemia (Chen et al., 2024). In addition, cellular autophagy has been found to have a significant impact on the prognosis of cerebrovascular disease. Studies have shown that appropriate autophagy can reduce neuronal death after SAH and improve cerebral edema and cerebral vasospasm (Zha et al., 2022; Liu et al., 2013). Intervention in autophagy is of great significance in the treatment of cerebrovascular diseases. In addition to the above mechanisms, there are other molecular mechanisms, such as iron death that are interrelated and interact with each other and work together in the pathophysiologic process of cerebrovascular diseases.

## 3 Biological basis of P2X7R

### 3.1 Structure and electrophysiology

P2 receptors belonging to purinergic receptors can be divided into two categories: P2X (with the ligand-gated cation channel P2X family consisting of P2X1-7) and P2Y coupled with G proteins (Zou Y. et al., 2023). The purinergic P2X7R is an important member of the P2X family. The activation of P2X7R, especially in microglia, can drive the activation of inflammatory pathways (Garrosa-Jiménez et al., 2021; Bhattacharya and Biber, 2016). P2X7R is mainly expressed in glial cells of the nervous system and macrophages and monocytes of the immune system (Qi et al., 2024; Zou L. et al., 2023).

**FIGURE 1:**
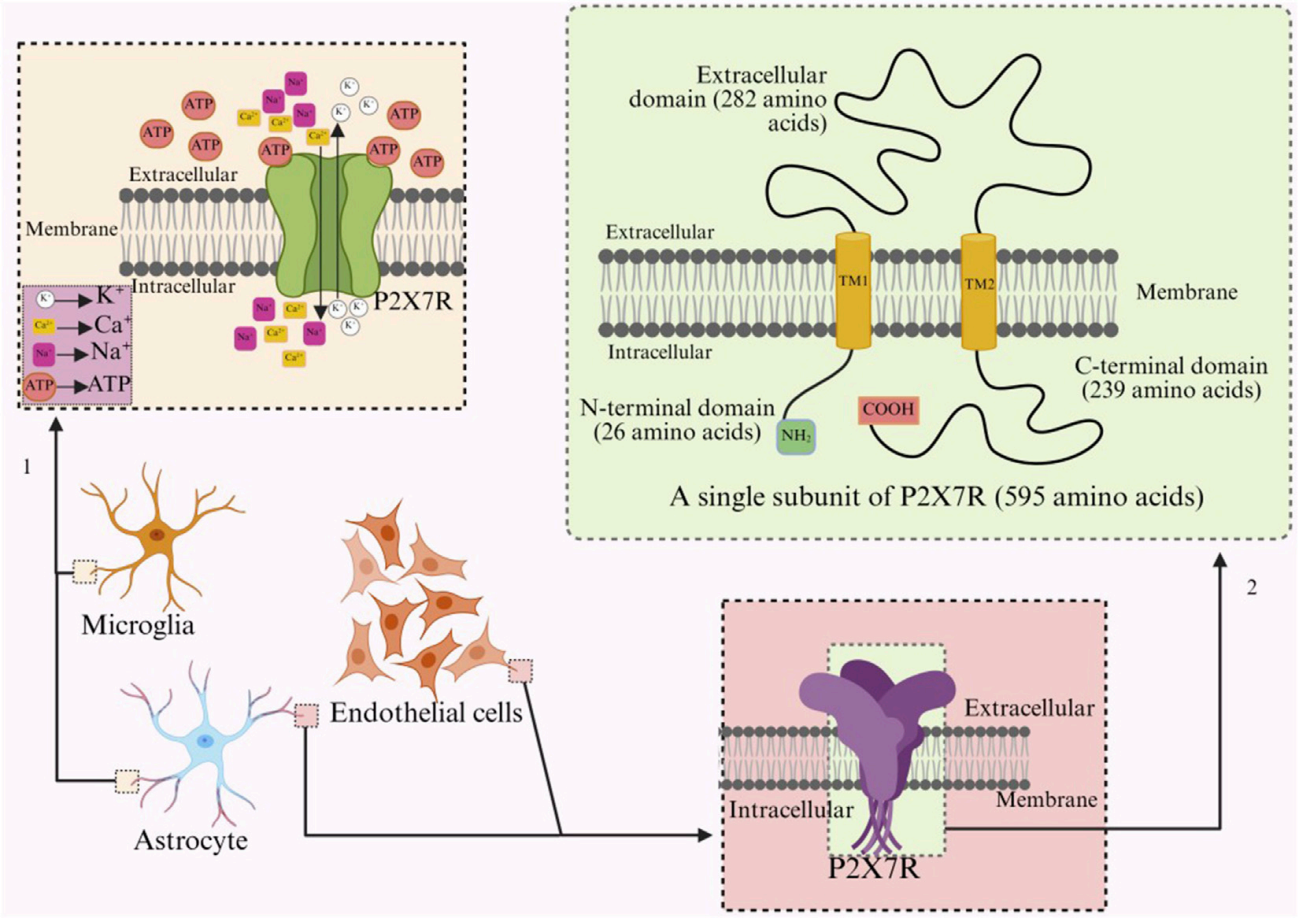
Arrow 1 refers to the ion transport of activated P2X7R on microglia, while arrow 2 refers to the structure and number of amino acids of a single subunit of P2X7R.

P2X7 is a membrane receptor composed of 3-6 subunits, and unlike other receptors in the P2X family that contain 379–472 amino acids per subunit, P2X7R increases to 595 amino acid residues (Sikka et al., 2022). Furthermore, P2X7R can form homotrimers and hexamers, but homotrimers are the dominant form of P2X7R *in vivo* (Aprile-Garcia et al., 2016). Functional P2X7R is composed of three identical subunits, similar to other subforms of the P2X receptor, with each subunit having an extracellular ring (282 amino acids), two transmembrane regions (TM1, TM2, 24 amino acids), an intracellular amino terminal (N-terminal, 26 amino acids) and a carboxy terminal (C-terminal, 239 amino acids) (Figure 1) (Zhang et al., 2023; Illes et al., 2019). The adenosine ATP binding site is located at the interface between two adjacent subunits (Urbina-Treviño et al., 2022; De Salis et al., 2022).

However, what is most striking is the intracellular C-terminal domain, which accounts for 40% of the entire subunit in size (Zhang et al., 2023; Wu et al., 2023). The unique structural characteristics of the C-terminus can modulate the function of P2X7R, including the activation of downstream signaling pathways such as the extracellular signal-regulated kinase (ERK) pathway and caspase-3 activation, thereby initiating apoptosis (Sun BX. et al., 2023). In addition, it may modulate functions such as receptor cell localization, protein-protein interactions, and post-translational modifications (Zhang Y et al., 2022; El Idrissi et al., 2024; Ortega et al., 2021). In addition, chimeric receptors combining the human extracellular domain and the rat C-terminus exhibit an inactivation time increase similar to that of the rat P2X7R, suggesting that the difference in the human C-terminus compared to the rat C-terminus may be the reason why human P2X7R inactivates faster than rat P2X7R (Urbina-Treviño et al., 2022; Bartlett et al., 2014).

P2X7R is an ATP-dependent, non-selective cation channel that is an important receptor for driving inflammation (von Muecke-Heim et al., 2021). ATP is not only a molecule that can directly supply energy, but also an extracellular signaling molecule that can affect many physiological and pathological responses, and extracellular ATP (eATP) reaching a certain concentration can activate P2X7R to participate in the release of inflammatory mediators (Müller et al., 2022; Huang and Tan, 2021; Du et al., 2015). P2X7R has a lower affinity for ATP than other P2X ligand-gated channels [semi-maximal effective concentration (EC50): 0.1–10 μM of ATP] and requires a high concentration of ATP (EC50: 2–4 mM) for activation (Urbina-Treviño et al., 2022; Lee et al., 2023). Moreover, the affinity of P2X7R for ATP is species-specific, and the concentrations of BzATP and ATP required for activation of human P2X7R (EC50: 20 μM and 100 μM, respectively) are lower than those of activated mouse P2X7R (EC50: 295 μM and 850 μM, respectively), and the affinity of human receptors for ATP is higher than that of mouse receptors (Urbina-Treviño et al., 2022). Therefore, under normal physiological conditions, P2X7R remains “silent” at eATP concentration levels in the low millimolar range, but under pathological conditions such as harmful stimuli, cell damage, and stress, the body will release a large amount of ATP (>100 μmol/L) to activate P2X7R (Wu et al., 2023; Lee et al., 2023), which can promote neurotransmitter release, neuroimmune dysregulation, neuroinflammatory response, and also enhance the innate immune response by inducing the proliferation and activation of some immune cells (Urbina-Treviño et al., 2022; Carmo et al., 2014; Clark et al., 2010; Monif et al., 2016).

ATP or an appropriate amount of the synthetic agonist BzATP binds to P2X7R, and the receptor conformation changes to open ion channels (Beltran-Lobo et al., 2023a; von Mücke-Heim et al., 2023), allowing Na^+^, Ca^2+^ to enter and K^+^ to flow out (Figure 1) (Zheng et al., 2024). The efflux of potassium reduces the intracellular K^+^ concentration, triggering inflammasome effects and the release of pro-inflammatory cytokines, such as the release of IL-1β and IL-6 from microglia and dendritic cells of different species (Zou L. et al., 2023; Zhang Y et al., 2022), and the change of intracellular ion concentration over time leads to a gradual change in equilibrium potential (Bhattacharya and Biber, 2016; Zhang Y et al., 2022; Simões et al., 2023). The formation of pore channels in P2X7R will gradually increase the amplitude of the ion current, but the inactivation rate will be slower (Cisneros-Mejorado et al., 2015b). The amplitude of the current through P2X7R is inversely related to the concentration of external divalent cations. The receptor is highly sensitive to changes in extracellular divalent cation concentrations. During pathological high-frequency nerve discharge, Ca^2+^ penetrates the cell through the pores formed by P2X7R, significantly reducing extracellular calcium ions (potentially by up to 90%) (Barros-Barbosa et al., 2016). This reduction further activates the receptor, greatly enhancing the influx of Na^+^. This influx may diminish the driving force for the transport of γ-aminobutyric acid and glutamate, which is dependent on the Na^+^ concentration gradient. Consequently, this process can disrupt neurotransmitter balance and contribute to neuroinflammatory and excitotoxic conditions (Barros-Barbosa et al., 2016; Ren et al., 2023). Under long-term or repeated stimulation of ATP or BzATP, P2X7R does not desensitize, but forms non-selective macropores that allow hydrophilic macromolecules with molecular weights up to 900 Da to pass through the cell membrane, and the passage of continuous currents can disrupt cellular homeostasis and even induce cell death (Bhattacharya and Biber, 2016; Simões et al., 2023; Surprenant et al., 1996).

### 3.2 Expression profile in the CNS and vasculature

Glutamatergic neurons within the hippocampal CA3 region have been reported to express the P2X7R, but neuronal expression of P2X7R is still under discussion (Beltran-Lobo et al., 2023a; Ortega et al., 2021; Metzger et al., 2017). In the human or rodent brain, P2X7R is expressed in glial cells (microglia, astrocytes, oligodendrocytes) in various brain regions, including the hippocampal striatum, neocortical white matter 1 (Wei et al., 2016; Li-yan et al., 2009; Bedetta et al., 2025; Sun et al., 2025). P2X7R was also present on pericytes of cerebral microvessels in rats undergoing experimental autoimmune encephalomyelitis (Gryg et al., 2018). In addition, P2X7R expression was also seen on endothelial cells 24 h after ICH (Zhao et al., 2016; Sun BX. et al., 2023). P2X7R is expressed in a variety of cell types in the brain, with higher levels in activated microglia than in other cell types (Beltran-Lobo et al., 2023a; Van Weehaeghe et al., 2019).

### 3.3 Regulation of P2X7R expression in the brain

#### 3.3.1 Microenvironmental stimulation (ischemia, brain injury) and ATP release

The expression of the P2X7R changes dynamically with the progression of neurological diseases and is associated with microglial activation (Zhao et al., 2016; Zou L. et al., 2023). Microglia are a specialized type of macrophage in the central nervous system (CNS) (Zhao et al., 2017; He et al., 2025). During neuroinflammation caused by events such as traumatic brain injury or stroke, cells may release high levels of ATP due to ischemia and hypoxia, and this excess ATP can activate P2X7R, which in turn activates glial cells and enhances the expression of P2X7R. This coexists with microglial activation (Zhao et al., 2016; Huang et al., 2017; Ollà et al., 2020; Wang M. et al., 2020; Qin et al., 2023). Microglia release various pro-inflammatory substances that act in an autocrine manner, promote the further activation of microglia, and form a harmful cycle of neuroinflammation via the opening of the P2X7R channel (Bedetta et al., 2025; Monif et al., 2010). Astaxanthin can rebalance intracellular and eATP concentrations, downregulate P2X7R expression, block harmful cycles, and relieve P2X7R-mediated microglial inflammation (Wang M. et al., 2020). A study using a rat model of middle cerebral artery occlusion found that P2X7R expression was upregulated in microglia, and subsequently upregulated in astrocytes and neurons (Lämmer et al., 2011).

#### 3.3.2 Expression regulatory factors (HIF-1α, Sp1, CpG methylation)

Another study using a mouse model of middle cerebral artery occlusion found that mild ischemic preconditioning caused a rapid and transient increase of the hypoxia-inducible factor 1α (HIF-1α) in neurons, followed by a slow and sustained increase in astrocytes, with the temporal pattern of P2X7R expression corresponding to that in astrocytes (Hirayama and Koizumi, 2017). Activation of multiple signaling pathways downstream of the P2X7R can produce molecules that induce HIF-1α expression, such as phosphoinositide 3-kinase, protein kinase B, through phosphorylation of ribosomal protein S6 kinase and eukaryotic translation initiation factor 4E-binding protein-1 increases the transcriptional activity of HIF-1α and promotes the conversion of HIF-1α mRNA to protein (Hirayama and Koizumi, 2017; Zhang et al., 2025). P2X7R promotes the release of HIF-1α and astrocytes develop ischemic tolerance (Ahn et al., 2023). In addition, the 5′-proximal regulatory region of the mouse P2X7R gene is speculated to contain regulatory elements that bind to HIF-1α, and it is possible that HIF-1α can directly regulate the expression of the P2X7R gene after binding to the corresponding locus (García-Huerta et al., 2012).

Neuroinflammation can lead to upregulation of specific protein 1 (Sp1) by activating the intracellular kinase cascade and upregulation of P2X7R in a transcription factor Sp1-dependent manner (Francistiová et al., 2020). In mouse models of status epilepticus, it was also found that the Sp1 transcription factor could promote P2X7R transcription in nerve cells (Rodriguez-Alvarez et al., 2017; Wei et al., 2024). In addition to neurons, Sp1 overexpression has also been found in mouse neuroma cells and mononuclear macrophage leukemia cells to increase gene promoter activity, and Sp1 binding to SP1c and SP1d sites helps recruit RNA polymerase II to the promoter, resulting in significantly elevated levels of endogenous P2X7 mRNA and protein (García-Huerta et al., 2012).

Downstream of the active promoter of the human P2X7R gene, it contains multiple cytosine-phosphodiester-guanosine (CpG) sites in the +26 to +573 nt region (Zhou et al., 2009). Several CpG sites downstream of the active promoter are direction-dependent cis elements of P2X7R transcription, which can regulate P2X7R gene transcription through changes in cytosine methylation status, hypermethylated CpG inhibits transcription, and demethylation increases transcription (Zhou et al., 2009). Hypermethylated CpG inhibits P2X7R transcription by modulating the interaction of enhancer transcription factors with their homologous deoxyribonucleotide acids (DNA)-binding domains, possibly by altering the spatial conformation of transcription factor recognition sites within the enhancer region (Zhou et al., 2009).

#### 3.3.3 Spliced variants (P2X7A, P2X7B)

The P2X7R gene has 13 exons that can be alternatively spliced and mutated to form multiple receptor subtypes. P2X7A is an mRNA encoding an integral protein, and the P2X7A isoform is a native isoform expressed in a variety of mammals (Zhang et al., 2023; Andrejew et al., 2020). Insertion of a new exon or absence of one or more genetic regions results in other splicing isoforms, such as the presence of an intron between P2X7B exons 10 and 11, and mutations that stop codon insertion occur, resulting in the P2X7B variant lacking a C-terminal long tail, resulting in a shortened P2X7 receptor that does not form a large pore that induces cell death (Zhang et al., 2023; Andrejew et al., 2020; Mishra et al., 2021).

#### 3.3.4 Regulation mechanisms of non-coding RNAs such as microRNAs

MicroRNAs that are non-coding RNAs and 19–24 nt in size regulate the expression of the P2X7R gene in cells after transcription (Ahn et al., 2023; Rodriguez-Alvarez et al., 2017; Jimenez-Mateos et al., 2015). The microRNA-22 promoter contains a specific Sp1 binding site (Jimenez-Mateos et al., 2015), and microRNA-22 can often target inhibition of P2X7R transcript translation (Ollà et al., 2020; Francistiová et al., 2020).

#### 3.3.5 Post-translational modifications (glycosylation, palmitoylation)

Human P2X7R is glycosylated at five residues of the N-chain (187, 202, 213, 241, and 284), and this post-translational modification is important for P2X7 agonist-stimulated signaling and P2X7R pore formation (Lenertz et al., 2010). Palmitoylation is a post-translational modification that plays an important role in the regulation of P2X7R. Palmitoylation refers to the covalent attachment of the 16-carbon fatty acid palmitate to proteins, usually with thioester bonds, and the palmitic acid bonds are reversible. Palmitoylation in the carboxy-terminal region of P2X7R allows for proper maturation of P2X7R (Gonnord et al., 2009; Li et al., 2025). In addition, P2X7R prevents P2X7R A desensitization by “anchoring” the cytoplasmic portion of TM2 to the membrane by the C-cys anchor containing at least five palmitoylated residues (McCarthy et al., 2019).

In summary, the underlying mechanism that controls P2X7R expression in the brain is likely to occur at the transcriptional level, but further research is needed on its specific mechanisms.

## 4 Pathological roles of P2X7R in cerebrovascular diseases

**FIGURE 2:**
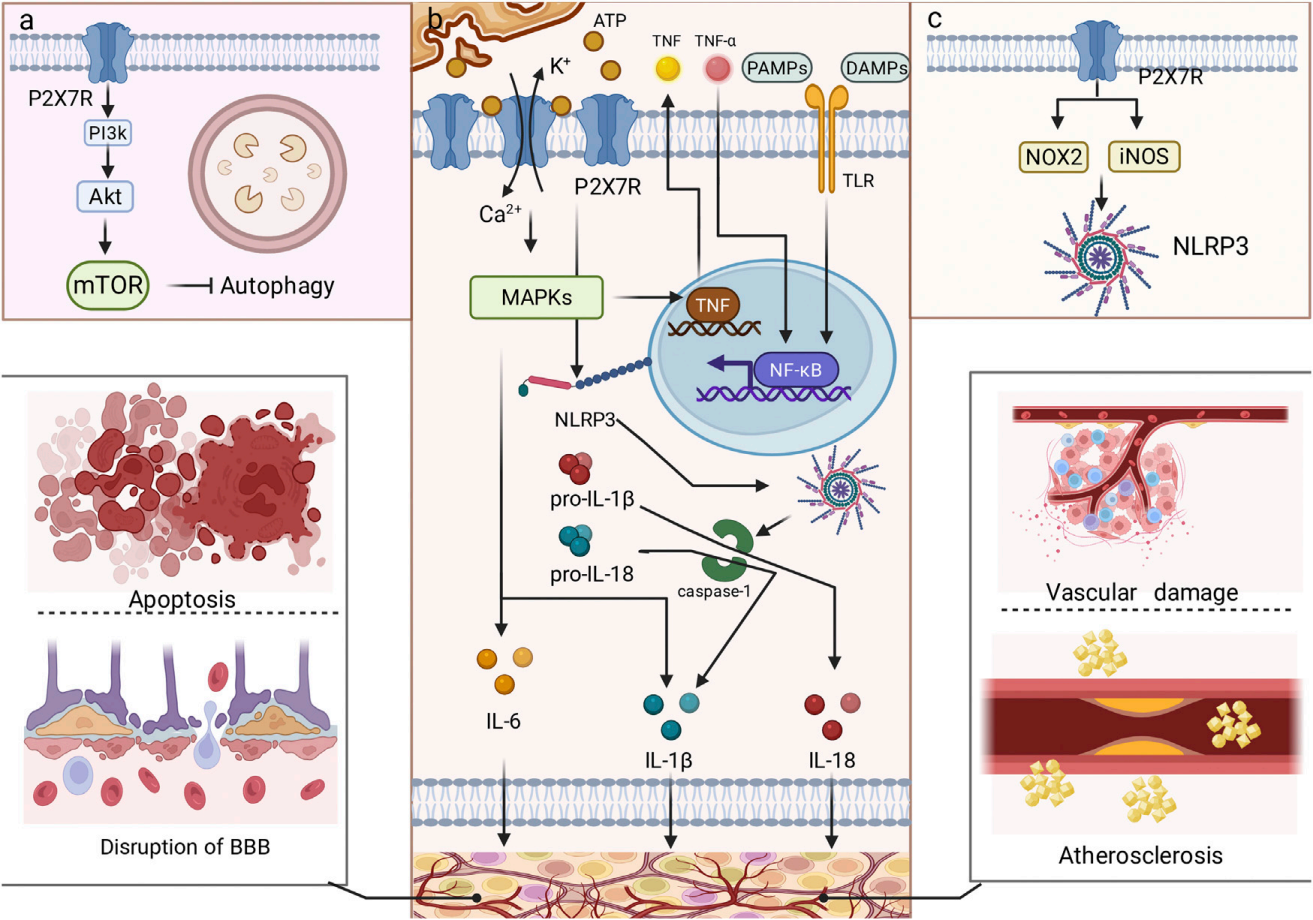
Common mechanisms involving P2X7Rs in cerebrovascular diseases. P2X7R is mainly involved in the development of cerebrovascular diseases through neuroinflammation, oxidative stress, and cellular autophagy. **(a)** shows cellular autophagy, **(b)** shows neuroinflammation, and **(c)** shows oxidative stress. Neuroinflammation is the most important molecular mechanism, which includes NLRP3, NF-κB, and MAPK pathways. P2X7R mediates apoptosis, disruption of BBB integrity, vascular damage, and atherosclerosis through these molecular mechanisms.

With the continuous research on the pathogenesis of various cerebrovascular diseases, it has been found that P2X7R may be involved in the occurrence and development of cerebrovascular diseases through neuroinflammation, oxidative stress and other mechanisms, and Figure 2 shows some of the mechanisms involved. P2X7R may be a therapeutic target for cerebrovascular diseases. To uncover the therapeutic potential of P2X7R in cerebrovascular disease, we reviewed and summarized research evidence linking P2X7R to various important cerebrovascular diseases.

### 4.1 P2X7R in SAH

Subarachnoid hemorrhage occurs when a blood vessel in the brain ruptures, causing blood to enter the subarachnoid space. It is an acute, poorly prognostic cerebrovascular disease. Early brain injury (EBI) and delayed brain injury (DBI) are the two primary pathological mechanisms of subarachnoid hemorrhage (SAH). In the past, researchers primarily focused on DBI, but recently, EBI has also garnered significant attention. Both are believed to be closely associated with the prognosis of SAH. A series of pathological changes occurring within 72 h after the onset of SAH, such as increased intracranial pressure, cerebral edema, blood-brain barrier damage, and neuronal death, are referred to as EBI (Zhang et al., 2020; Zhou et al., 2021). Its occurrence is primarily due to the physiological and pathological damage caused by a large amount of blood and its metabolic byproducts entering the subarachnoid space. EBI significantly impacts patient disability rates and mortality, and intervention targeting EBI is considered a crucial therapeutic approach for SAH. DBI is an important pathophysiological event occurring 3–14 days after SAH. During this period, delayed cerebral ischemia caused by cerebral vasospasm, microthrombosis, and diffuse cortical ischemia is an important cause of DBI and also an important factor influencing patient prognosis (Macdonald, 2014). In addition, EBI also has an impact on the occurrence of DBI (Kumagai et al., 2020). Current research focuses on investigating the mechanisms underlying these two types of brain injury, with the aim of achieving therapeutic effects and improving outcomes by inhibiting and improving EBI and DBI following SAH. P2X7R has been found to potentially exert therapeutic effects by influencing EBI and DBI following SAH.

The inflammatory pathway is the main pathway through which P2X7R affects brain damage after SAH. To understand this pathway, it is first necessary to understand the “two-step” theory of the NLRP3 inflammasome and the role of P2X7R in it. NLRP3 inflammasome is able to activate caspase-1, which in turn is able to activate pro-inflammatory cytokines such as IL-1β and IL-18. The activation of NLRP3 inflammasome consists of two steps: “initiation” and “activation”. During the initiation phase NLRP3, pro-IL-1β and pro-IL-18 are expressed and translated to gain the ability to respond to activating factors in preparation for the second step, “activation.” At this point, NLRP3 complexes do not have the ability to activate caspase-1 (Hafner-Bratkovič and Pelegrín, 2018). In the first step, the TLRs/nuclear factor-κB (NF-κB)/NLRP3 pathway has been found to be relevant to this process. TLRs are able to activate NF-κB through activation of pathogen-associated molecular patterns and injury/risk-associated molecular patterns, and they can also directly activate NF-κB by upregulating the expression of transmembrane pro-tumor necrosis factor (TNF)-α, which is cleaved by TNF-α convertase to form soluble TNF-α (Li et al., 2021; Barberà-Cremades et al., 2017). NF-κB upregulates the expression of NLRP3, pro-IL-1β and pro-IL-NF-κB upregulates the expression of NLRP3, pro-IL-1β and pro-IL-18, and the second step is the assembly of inflammasome and the activation of caspase-1, which leads to auto-proteolytic activation of caspase-1, ultimately resulting in the cleavage of proIL-1β and proIL-18 to reactive IL-1β and IL-18. These inflammatory factors are released by the cell, such as through the formation of transmembrane pores or vesicles, and ultimately trigger a series of inflammatory responses (Mishra et al., 2021; Muñoz-Planillo et al., 2013; Leavy, 2013; Xia GQ. et al., 2022). Notably, IL-1β is scarcely present at baseline and requires an inducing stimulus (e.g., LPS) for expression, which is unnecessary for IL-18 release (Mishra et al., 2021). The canonical NLRP3 inflammasome activation depends on caspase-1, while the non-canonical pathway relies on caspase-11 and caspase-4. Caspase-11 and caspase-4 promote the release of IL-1β and IL-18, leading to pyroptosis, and also assist in the activation of caspase-1 (Xiang et al., 2020). Canonical and non-canonical NLRP3 inflammasome activation is dependent on K^+^ efflux. Activation of P2X7R by millimolar concentrations of extracellular ATP mediates Na^+^ and Ca^2+^ efflux and K^+^ efflux, leading to a decrease in intracellular K^+^ and thus NLRP3 inflammasome activation (Hafner-Bratkovič and Pelegrín, 2018). The above process is involved in the inflammatory response and outcome of many diseases, and is also present in SAH.

Under stress and injury conditions, cells release various intracellular molecules, such as ATP, to activate inflammatory responses and repair damaged tissues. These molecules are called damage-associated molecular patterns (DAMPs) (Ahmed et al., 2021). After SAH occurs, DAMPs can activate TLRs, followed by the activation of P2X7R by extracellular ATP, which synthesizes and releases various inflammatory substances (such as TNF-α, IL-1β, IL-6, etc.) and matrix metalloproteinase (MMP)-9 through pathways such as NF-KB and Mitogen-activated protein kinases (MAPK), thereby exacerbating brain tissue damage after SAH (Okada and Suzuki, 2020; Ming et al., 2024; Tian et al., 2022; Nishikawa et al., 2018; You et al., 2013). The P2X7R/MAPK signaling pathway is closely related to the regulation of inflammatory factors and the progression of SAH. MAPK is a group of serine-threonine kinases, also known as mitogen-activated protein kinases, which can be activated by extracellular stimuli (such as lipopolysaccharide (LPS)) and regulate various physiological processes (Gehart et al., 2010). MAPK comprises numerous subfamilies, among which the inflammatory response regulated by ERK, Jun N-terminal kinase (JNK), and p38 kinase is associated with P2X7R. In LPS-induced BV2 microglia, the phosphorylation levels of p38 MAPK, JNK, and ERK1/2 are upregulated, and the expression of IL-1β, IL-6, and TNF-α is increased, while the antagonist Brilliant Blue G (BBG) can improve this situation (Wang W. et al., 2020). In another experiment, after applying the P2X7R antagonist BBG, the phosphorylation level of p38 MAPK decreased, the activation of microglia was inhibited, the expression of proinflammatory factors was reduced, and ultimately neuronal damage was alleviated (Wang et al., 2017; Choi et al., 2007). It is also worth noting that the process by which P2X7R activates microglia to release TNF is regulated by several pathways in MAPK. Protein tyrosine kinase is located downstream of P2X7R and can activate JNK and p38 MAPK. ERK and JNK participate in the regulation of TNF mRNA expression, while p38 MAPK participates in the ribosomal transport of TNF mRNA (Suzuki et al., 2004).

The IL-1β, IL-6, TNF-α, and nitric oxide synthase (iNOS) produced by P2X7R regulation were found to be associated with the severity of cerebral vasospasm in SAH. Inhibiting these inflammatory factors can reduce neuronal apoptosis, cerebral vasospasm, and blood-brain barrier disruption, thereby alleviating brain damage after SAH (Hong et al., 2023; Ding et al., 2014; Kuai et al., 2022). Research has found that heat shock protein 90 activation reduces levels of the neurotrophic factor BDNF and diminishes neurogenesis by mediating P2X7-dependent IL-1β release, thereby participating in EBI following the pathological process of SAH (Zuo et al., 2018). Moreover, P2X7-dependent IL-1β release can induce the secretion and activation of MMP-9 through the NF-κB signaling pathway. Proinflammatory cytokines and MMP-9 can disrupt the blood-brain barrier and promote apoptosis, thereby exacerbating EBI after SAH (Sun XG. et al., 2019; Simon and Grote, 2021). The destruction of the blood-brain barrier by MMP-9 leads to the release of various inflammatory factors by macrophages and neutrophils, causing cerebral vasoconstriction and cerebral edema (Guo et al., 2010; Mehta et al., 2013). It is worth noting that MMP-9 can damage the alveolar barrier and ultimately lead to neurogenic pulmonary edema, a serious complication following SAH (Chen et al., 2014). The P2X7R inhibitor BBG can suppress the associated inflammatory response, thereby improving tissue damage following SAH (Chen et al., 2014; Chen et al., 2013). Similarly, a recent study found that the With No lysine (K) kinase 1 can alleviate neurological dysfunction following subarachnoid hemorrhage by inhibiting P2X7R- related NLRP3 inflammasome activation (Zhao et al., 2025). In conclusion, modulating P2X7R can reduce the impact of inflammatory responses on tissues, thereby potentially exerting a therapeutic effect on brain damage following SAH.

In addition to the neuroinflammatory mechanism, P2X7R also influences brain damage after SAH by regulating autophagy, which is another noteworthy mechanism. Autophagy is a complex process that, under physiological conditions, breaks down and metabolizes unnecessary cellular components and plays a role in maintaining cellular balance and protecting cells. However, under pathological conditions, autophagy dysfunction may lead to damage. Numerous studies have investigated the effects of autophagy-related pathways in neurons on EBI and DBI following SAH, finding that appropriate autophagy can exert a protective effect, but excessive autophagy promotes brain injury after SAH. Autophagy can improve blood-brain barrier (BBB) permeability and ultimately reduce brain edema after SAH by regulating the death of vascular endothelial cells (Wang et al., 2012).

A study on autophagy and DBI after SAH found that autophagy activation can improve cerebral vasospasm, thereby improving DBI (Liu et al., 2013). In addition, enhancing autophagy function during the EBI period after SAH can also prevent DBI (Sun C. et al., 2019). Autophagy activated and enhanced through various means can reduce neuronal death, improve neurological function after SAH, and exhibit neuroprotective effects (Zha et al., 2022; Guo et al., 2018). However, studies have found that FGF-2 can exert neuroprotective effects in EBI after SAH by inhibiting autophagy after SAH (Wang Y. et al., 2021). The abovementioned research confirmed the dual role of cellular autophagy in SAH. The focus then shifted to P2X7R. Research found that SAH led to increased expression of DNMT1, which increased the methylation level of the promoter containing the Milk fat globule EGF and factor V/VIII domains of milk fat globule epidermal growth factor like 8 (MFGE8), thereby MFGE8. The downregulation of MFGE8 leads to the overexpression of P2X7R, which subsequently reduces autophagy by inhibiting the PI3K/Akt/mTOR axis, ultimately exacerbating EBI following SAH (Zou L. et al., 2023; Kim et al., 2019). Inhibiting P2X7R can increase autophagy through the PI3K/Akt/mTOR pathway, thereby exerting a neuroprotective effect (Zou L. et al., 2023).

Overall, P2X7R mainly acts through neuroinflammation and autophagy in brain damage after SAH, with inflammation being the main pathological mechanism. Studies have shown that inhibiting P2X7R can improve EBI and DBI after SAH, suggesting the potential therapeutic value of P2X7R.

### 4.2 P2X7R in intracerebral hemorrhage

Brain edema and disruption of the blood-brain barrier, the main pathological changes leading to a poor prognosis in ICH, arise and develop in close association with inflammation as well as oxidative stress. After the rupture of a cerebral blood vessel, a large number of erythrocytes and their metabolites (including hemoglobin), coagulation factors, and complement components enter the brain parenchyma, activating inflammatory and oxidative stress responses that cause tremendous cellular damage, a process that leads to cerebral edema and destruction of the BBB, inducing tissue damage (Chen et al., 2022). Initial brain damage in ICH is caused by the mechanical disruption resulting from hematoma, which both destroys the BBB and also causes swelling of parenchymal cells, which induces cerebral edema (Yang et al., 1994; Xia F. et al., 2022). What follows is an inflammation activated by the release of various neurotoxins-coagulation factors, erythrocyte metabolites, and fibrinogen. These induced inflammatory responses damage cerebral microvascular endothelial cells and perivascular glial cells, further leading to BBB damage (Liu and Sharp, 2011). Activated microglia, astrocytes, and leukocytes in the region surrounding the hematoma are also capable of inducing a series of pro-inflammatory cascade responses that lead to neuronal cell death. The increase in inflammatory factors and activation of glial cells in the perihematomal region is also an important cause of the destruction of the BBB (Ren et al., 2020). In addition, iron overload in the perihematomal region induces ROS with lipid peroxidation that destroys intracellular proteins and nucleic acids, ultimately leading to cell death (Wan et al., 2019). Oxidative stress and inflammatory response in the area surrounding the hematoma are important causes of tissue damage after ICH. The presence of activated microglia, astrocytes, and peripheral immune cells in the area surrounding the hematoma mediates the inflammatory response (Ren et al., 2020). In addition, there is increased expression of various inflammatory mediators in the region, including cytokines such as IL-1, IL-18, TNF-α, and chemokines such as C-C motif ligand (CCL)2, CCL5, CCL17, CCL20, and MMP. P2X7R mediates these inflammatory components, causing BBB damage and cerebral edema, leading to poor prognosis of brain injury after ICH.

Following ICH, P2X7R is primarily expressed on astrocytes and endothelial cells. The Ras homolog family member A (RhoA), a downstream signaling molecule of P2X7R, is activated, thereby downregulating the expression of endothelial junction proteins and disrupting the integrity of the BBB (Zhao et al., 2016). In addition to disrupting the integrity of the BBB, P2X7R also contributes to secondary damage following ICH by inducing oxidative stress and neuroinflammation. Similar to SAH, cellular damage caused by ICH releases DAMP, which activates TLR and, together with the metabolic products of clotting factors and red blood cells that enter the brain parenchyma, mediates the inflammatory response around the hematoma (Wan et al., 2023). After ICH, increased production and secretion of P2X7-dependent IL-1β and IL-18 in activated microglia causes neuronal death, BBB disruption, and brain edema, resulting in poor ICH prognosis (Fang et al., 2024). After cerebral hemorrhage, activation of P2X7R can activate nicotinamide adenine dinucleotide phosphate oxidase (NOX)2 through the ERK1/2 and NF-κB pathways, thereby increasing NOX2-mediated oxidative stress levels and activating the NLRP3 complex (Deng et al., 2021; Yang et al., 2014; Hewinson et al., 2008). Additionally, P2X7R can induce the release of nitrite by simultaneously activating iNOS, thereby increased production and secretion of P2X7-dependent IL-1β and IL-18, ultimately resulting in neuronal death and inflammatory tissue damage (Feng et al., 2016; Xiao et al., 2020; Barker et al., 2011). Targeting the P2X7R/NLRP3 inflammasome pathway to improve secondary brain damage after ICH has been identified as a potential therapeutic target. Following ICH, endogenous H2S synthesis is reduced. By inhibiting the expression of P2X7R on microglia, supplementing brain H2S can alleviate damage caused by neuroinflammation associated with NLRP3 inflammatory responses following ICH (Zhao et al., 2017; Zhao et al., 2018).

P2X7R also plays an important role in the development of brain injury after ICH by activating various chemokine-mediated inflammatory responses. In microglia, P2X7R can promote the production of the cytokine CCL2 through the NF-κB signaling pathway (Beltran-Lobo et al., 2023b). CCL2 can disrupt the BBB by activating the p38 MAPK pathway, leading to cerebral edema and resulting in neurobehavioral dysfunction. Inhibition of the CCL2 receptor protects the integrity of the BBB and improves neurobehavioral deficits (Guo et al., 2020). CCR5, which is located on microglia, astrocytes, and monocytes, and its ligand CCL5, are overexpressed after ICH. Peripheral inflammation after ICH can activate the CCR5/CCL5 axis through P2X7R and upregulate the expression of MMP-9 through the classic inflammatory signaling pathway JAK2/STAT3. MMP-9 degrades tight junction proteins, thereby disrupting the integrity of the BBB and exacerbating cerebral edema. Inhibition of CCR5 can improve this phenomenon by inhibiting the activation of P2X7R (Sun W. et al., 2023; Lin et al., 2023). In addition, it has been found that the P2X7R agonist BzATP stimulates the release of CCL20, which plays an important role in the inflammatory mechanism of brain injury after ICH (Lim et al., 2016; Zheng et al., 2023).

Research indicates that P2X7R primarily participates in the pathogenesis of hemorrhagic stroke through inflammatory responses, and oxidative stress mechanisms are also involved. Inhibiting P2X7R may be effective in improving brain damage associated with ICH. Currently, there is no P2X7R antagonist treatment group in the ICH model. Further exploration of the mechanism is needed to ultimately discover more pathways related to the prognosis of ICH, thereby providing more treatment options for ICH patients.

### 4.3 Ischemic stroke

Stroke is a serious cerebrovascular disease with high morbidity, mortality, and disability rate, which has become one of the main diseases endangering human health and life, of which 80% are caused by ischemia. Ischemic stroke is not only one of the major complications of clinical emergencies such as cardiac arrest, drowning, or severe systemic hypotension during surgery, but it is also a leading cause of long-term disability (Sun et al., 2021). Reperfusion is by far the best treatment for the patient. However, this can exacerbate the damage to the brain, known as cerebral ischemia/reperfusion (I/R) injury (Chu et al., 2012). Due to ischemia, brain cells are deprived of oxygen, glucose, and energy, resulting in brain damage. Damaged cells trigger various pathophysiological processes, such as neuronal apoptosis, neuroinflammation, oxidative stress and glutamate excitotoxicity (Ri et al., 2023; Ye et al., 2018).

Neuronal apoptosis and inflammatory responses are more crucial in the pathogenesis of ischemic stroke, among which P2X7R plays a significant role. In the context of neuronal apoptosis, P2X7R activation in microglia promotes NLRP3 inflammasome assembly and the subsequent release of mature IL-1β. This inflammatory milieu, in turn, induces caspase-3 activation and apoptotic signaling in neurons. This indirect mechanism underscores that P2X7R exerts its pro-apoptotic effects not by acting within neurons, but primarily by driving a neurotoxic response from microglia—a principle consistent with findings that microglial P2X7R dysregulation directly compromises neuronal integrity (Huang S. et al., 2024). However, the downstream proteins of the P2X7R/NLRP3 pathway are not fully clear (Ye et al., 2017). In the inflammatory response, the increased expression of chemokines leads to the aggregation and adhesion of inflammatory cells, which not only destroys the integrity of the BBB endothelial cells and microvessels but also increases the BBB permeability (Bai et al., 2021). Glia-induced nerves can also disrupt the blood-brain barrier and increase bleeding transformation in ischemia-induced tissue injury and reperfusion injury. Inflammation is one of the main defense functions of glial cells, which are originally used to clear debris from brain injury and repair damaged tissues, but glial cells can cause excessive inflammation and thus increase neuronal mortality (Chu et al., 2012). In ischemic stroke, the inflammatory response can activate astrocytes and microglia to release inflammatory factors, and it also increases the expression of P2X7R in astrocytes and microglia. Under normal circumstances, the extracellular ATP concentration is insufficient to activate P2X7R. However, in ischemic stroke, the extracellular ATP concentration increases to activate P2X7R, and then calcium ions flow into microglia to activate second messengers, such as protein kinase C, phosphatidylinositol 3-kinase, phospholipase C, and MAPK. These will lead to an increase in the release of pro-inflammatory cytokines and other inflammatory mediators, thereby promoting the inflammatory response. Pro-inflammatory cytokines, such as interleukin IL-1β and IL-18, as well as the NLRP3 inflammasome, will be upregulated in expression (Ri et al., 2023). Following cerebral ischemia/reperfusion, the NLRP3 inflammasome is rapidly activated within microglia, serving as a primary source of inflammatory mediators such as IL-1β. This microglial response can initiate a feed-forward loop, further amplifying neuroinflammation and leading to secondary neuronal dysfunction (Ahn et al., 2023; Huang S. et al., 2024).

Oxidative stress refers to the excessive generation of peroxides and the depletion of antioxidants. Among them, the excessive ROS are the root cause of oxidative stress. Among them, the ROS derived from NOX play a crucial role in oxidative stress. Studies have shown that after cerebral ischemic injury, the levels of NOX2 and NOX4 significantly increase, generating excessive ROS, causing mitochondrial damage, and ultimately leading to neuronal death (Chen et al., 2024). Meanwhile, P2X7R activates NOX2 through the ERK1/2 and NF-κB pathways, thereby exacerbating oxidative damage. Additionally, the influx of Ca^2+^ that opens the mitochondrial permeability transition pore amplifies the release of ROS (Deng et al., 2021).

Glutamate excitatory toxicity is also considered to be an important cause of ischemic stroke (Zong et al., 2024). Glutamic acid is the main excitatory neurotransmitter in the brain. Glutaminase plays a key role in the preliminary metabolism of glutamine, which can catalyze the deamination of glutamine to produce glutamic acid and ammonia. Cerebral ischemia leads to upregulation of glutaminase and excessive accumulation of glutamate. However, glutamate excessively activates calcium inflow, increases intracellular calcium concentration, activates signaling cascade reaction, and induces neuronal injury and death through synergistic action (Wang J. et al., 2024). The Ca^2+^ influx mediated by P2X7R can enhance glutamatergic signal transmission (Barros-Barbosa et al., 2016), and the release of glutamate will further activate P2X7R, creating a vicious cycle (Zong et al., 2024).

In conclusion, P2X7R plays a role in inflammatory responses, oxidative stress, and glutamate excitotoxicity. Inhibiting the activation of P2X7R may lead to improvement, which provides a new therapeutic target.

### 4.4 Atherosclerosis

Atherosclerosis is a high-prevalence chronic disease worldwide, and most heart attacks and strokes are the result of atherosclerotic thrombosis, which is the leading cause of death and morbidity in many countries (Li et al., 2024). Atherosclerosis arises from the chronic inflammatory process of endothelial injury, followed by lipid deposition and plaque formation, which is caused by a combination of oxidative stress, impaired lipid metabolism, and inflammation (Zhu et al., 2024).

Oxidized low-density lipoprotein (oxLDL) is a key factor in the initiation of atherosclerosis. The presence of reactive oxygen species can lead to lipid peroxidation reactions. Oxidized low-density lipoprotein not only causes the formation of inflammation-mediated atherosclerosis but also forms oxidative-specific epitopes (OSE), which are considered immunogenic and have a pro-atherosclerotic effect. Studies have shown that oxidized phospholipids or malondialdehyde epitopes are recognized by scavenger receptors on macrophages (SRA, cluster of differentiation (CD) 36, and LOX-1), thereby increasing the uptake of oxLDL and forming foam cells. Additionally, OSE, as a risk-related molecular pattern, is recognized by pattern recognition receptors (such as CD36 and Toll-like receptor 4 (TLR4)), which subsequently releases P2X7-dependent IL-1β, and then promotes the maturation and release of caspase-1-mediated IL-1β, ultimately leading to an inflammatory response, resulting in endothelial cell activation, vascular smooth muscle cell proliferation, and the recruitment of lymphocytes and monocytes, as well as the secretion of pro-inflammatory cytokines (Peng et al., 2015; Li et al., 2024; Wang Y. et al., 2024). In this procedure, P2X7R is an essential bridge element. By upregulating P2X7R, OxLDL increases PKR phosphorylation and speeds up the NLRP3 inflammasome’s activation. P2X7R also mediates the release of MMP-9, which weakens the fibrous cap of the plaque and raises the possibility of rupture (Wang Y. et al., 2024; Lin et al., 2018). Intracellular oxLDL accumulation promotes ROS accumulation, which increases the apoptosis of vascular endothelial cell (VEC) and the expression of adhesion factors, thereby exacerbating the development of atherosclerosis (Mu et al., 2024).

Disorders of lipid metabolism are another significant contributor to atherosclerosis. Research has indicated that P2X7R may contribute to disruption in lipid metabolism via controlling the CXC chemokine ligand (CXCL16) pathway. CXCL16, one of the few clearance receptors with two distinct forms (membrane-bound and soluble), was first identified as a receptor for phosphatidylserine and oxidized low-density lipoprotein. When P2X7R is activated, the membrane-bound CXCL16 attaches to oxLDL and endocytoses it, accelerating the internalization process and encouraging the production of foam cells (Sheikine and Hansson, 2004). The soluble form of CXCL16 is produced through proteolytic cleavage by a disintegrin and metalloproteinase (ADAM) 10 and ADAM17, and it functions as a chemotactic factor in cells expressing CXCR6 (such as natural killer T cells and polarized helper T cells) (Aslanian and Charo, 2006). The expression of CXCL16 and CXCR6 is increased in human carotid plaques, while the expression of ADAM10 is decreased, which leads to an increase in podocyte uptake of oxLDL compared to normal veins and arteries. Simultaneously, activation of P2X7R results in rapid shedding of CXCL16, indicating that activation of P2X7R during chronic inflammation affects the CXCL16 pathway involved in foam cell formation. It has been reported that knocking out P2X7R increases the body weight and epididymal fat pad weight of mice and reduces plasma total cholesterol levels, suggesting that P2X7R plays a key role in regulating lipid storage and metabolism in the body. Therefore, knocking out P2X7R may delay the *in vivo* progression of atherosclerosis (Peng et al., 2015; Wang Y. et al., 2024). ATP is considered a major trigger of atherosclerosis. P2X7R, as a receptor of ATP, can mediate ATP-induced inflammatory response in macrophages. P2X7R is the main mediator in the process of ATP-induced cytokine secretion, which activates NLRP3 and caspase-1 and secretes IL-1β. In contrast to other P2X channels, because P2X7R does not reduce sensitivity during ATP stimulation, P2X7R can remain activated for a longer period of time, leading to the generation of ATP-induced inflammation. When atherogenesis occurs, ATP is released into the extracellular matrix, leading to extensive inflammation and monocyte migration (Tian et al., 2020). Therefore, P2X7R is thought to be involved in lipid accumulation primarily through its inflammatory phenotype (Hu et al., 2016).

Macrophages, as the most important inflammatory cells in the lesion, exhibit high plasticity and can differentiate into pro-inflammatory or healing-promoting forms upon stimulation by various factors. The pro-inflammatory macrophages can secrete cytokines such as IL-1β and IL-6. Meanwhile, the healing-promoting macrophages have been reported to significantly inhibit the formation of foam cells and reduce the development of atherosclerotic plaques. However, in this disease, activation of P2X7R leads to an imbalance in macrophage polarization, promoting the pro-inflammatory phenotype, inhibiting the healing-promoting phenotype, and accelerating the expansion of the necrotic core (Zhang Z. et al., 2022). Although the inflammatory response plays an indispensable role in the transition from a stable plaque state to an active plaque state, macrophages can accelerate cell necrosis, increase the necrotic lipid core area, and thicken the fibrous cap by degrading the plaque collagen. On one hand, when macrophages engulf ox-LDL, cholesterol crystals form in the cytoplasm and gradually transform into foam cells. This process activates P2X7R, promotes the assembly of the NLRP3 inflammatory body, and releases pro-inflammatory factors, accelerating cell overheating. Moreover, if these necrotic cells are not treated promptly, they will accumulate in the subendothelial layer, leading to an increase in the necrotic lipid core beneath the fibrous cap. On the other hand, macrophages can significantly increase the expression of MMPs, accelerate the degradation of the extracellular matrix (ECM), weaken the fibrous cap of the plaque, and promote plaque rupture (Huang R. et al., 2024; Poto et al., 2024).

In summary, targeted therapy of P2X7R cannot only block the assembly of the NLRP3 inflammatory body, thereby reducing inflammation, but also can inhibit the cleavage of CXCL16, reducing lipid accumulation. Moreover, it can reverse the imbalance of macrophage polarization, stabilize the plaque, and prevent its rupture.

## 5 Pharmacological modulation of P2X7R

**FIGURE 3:**
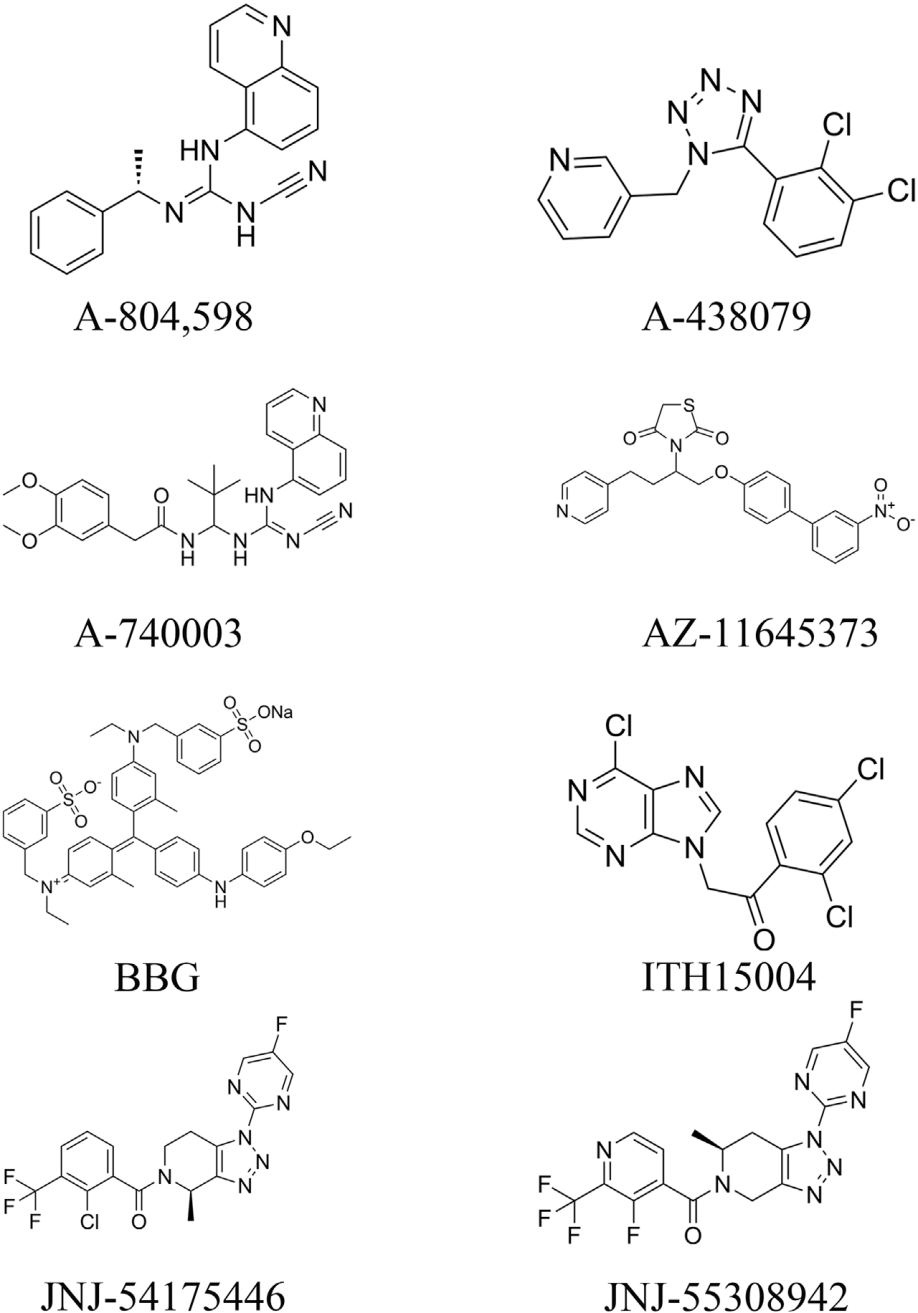
Molecular structures of currently available P2X7R antagonists.

From the existing studies, it is clear that P2X7R is a potential therapeutic target for cerebrovascular diseases. Pharmacological studies with P2X7R antagonists are of interest for the future treatment of cerebrovascular diseases. In this paper, we summarize the currently available P2X7R antagonists that are or may be related to cerebrovascular diseases and present them in Table 1 and Figure 3.

**TABLE 1:** P2X7R antagonists that are or may be related to cerebrovascular disease.

Antagonists	Chemical formula	Weight	Species	Antagonistic activity	References
A-804,598	C19H17N5	315.38	Human IC50 = 11 nM; Rat 1321N1 cells IC50 = 10 nM Kd = 2.4 nM Bmax = 0.56 pmol/mg; Mouse IC50 = 9 nM	A potential antagonist which can tolerate high concentrations in brain tissue and reduce the level of inflammatory markers	Hu et al. (2023), Donnelly-Roberts et al. (2009a)
AZ-11645373	C24H21N3O5S	463.51	Human THP-1 cells IC50 = 90 nM Kb = 92 nM	It is a highly selective and potent human P2X7R antagonist that inhibits the expression of inflammatory molecules	Bin et al. (2019), Oskolkova et al. (2017), Stokes et al. (2006)
JNJ-54175446	C18H13CIF4N6O	440.78	Human pIC50 = 8.46; Rat pIC50 = 8.81	It is a highly selective antagonist with good blood-brain barrier penetration and reduces the release of IL-1β from peripheral blood cells. Its *in vivo* target engagement ED_50_ of 0.46 mg/kg and the corresponding plasma EC_50_ of 105 ng/mL in rats	Illes et al. (2023), Letavic et al. (2017)
ITH15004	C13H7Cl3N4O	341.58	Human HEK293 cells IC50 = 9 µM (YO-PRO-1 uptake assay) IC50 = 13.7 µM (Intracellular Ca^2+^ dynamics assay)	It is a P2X7R antagonist with high lipophilic membrane permeability, which can reduce the release of IL-1β	de Pascual et al. (2021), Calzaferri et al. (2021)
JNJ-64413739			Human IC50 = 23 nM Kd ≈ 7 nM	It is a selective, metabolically stable, and brain permeable P2X7R antagonist. *In vivo*, ED_50_ is 14.52 mg, and the corresponding EC_50_ is 44.3 ng/mL	Mikkelsen et al. (2023), Koole et al. (2019)
JNJ-55308942	C17H12F5N7O	425.32	Human IC50 = 10 nM Ki = 7.1 nM; Rat IC50 = 15 nM Ki = 2.9 nM	It is a potent selective P2X7 antagonist with oral bioavailability that blocks IL-1β release from microglia in a concentration-dependent manner. *In vivo*, ED_50_ is 0.07 mg/kg(oral), EC50 brain is 12 ng/mL and EC50 Plasma is 15 ng/mL	Bhattacharya et al. (2018), Chrovian et al. (2018)
JNJ-54232334			Human pIC50 = 9.57 ± 0.02; Rat pIC50 = 7.57 ± 0.02 Kd = 4.9 nM Ki = 0.5 nM	It is an antagonist with a strong affinity for human P2X7R	Lord et al. (2015), Dal Ben et al. (2025)
Brilliant Blue G (BBG)	C47H48N3NaO7S2	854.02	Human IC50 = 265 nM; Rat IC50 = 10.1 nM	It is a selective antagonist, not only decreased IL-1β levels, but also reduce activation of astrocytes and microglia	Alves et al. (2023), Jiang et al. (2000)
A-740003	C26H30N6O3	474.55	Human pIC50 = 7.36 ± 0.01; Rat pIC50 = 7.74 ± 0.02	It is a selective P2X7R antagonist that reduces glutamate and IL-1β release	Ye et al. (2018), Gicquel et al. (2015), Donnelly-Roberts et al. (2009b)
A-438079	C13H9Cl2N5	306.15	Human pIC50 = 6.91 ± 0.01; Rat pIC50 = 6.51 ± 0.03	It is a selective P2X7R antagonist that crosses the blood-brain barrier, has a short half-life and low bioavailability, and reduces oxLDL accumulation in human podocytes	Donnelly-Roberts et al. (2009b), Genetzakis et al. (2022)

P2X7R, P2X7 Receptor; IC50, half maximal inhibitory concentration; Kd, equilibrium dissociation constant; Bmax, number of binding sites; Kb, antagonist affinity constant; pIC50, negative logarithm of the half maximal inhibitory concentration; ED_50_, 50% effective dose; EC_50_, 50% effective concentration; Ki, the affinity constant of drug-target binding; BBG, Brilliant Blue G; IL, Interleukin.

### 5.1 Advances in P2X7R antagonists and agonists

P2X7R is mainly activated by the high concentration of extracellular a ATP (Koo et al., 2017; Gil-Redondo et al., 2022). It also regulates Na+, Ca2+, K+ and other cations, thereby causing a variety of downstream cascades, such as mediating neuroinflammation, promoting the activation and assembly of NLRP3 inflammasome, triggering the maturation and release of IL-1β, and triggering cell apoptosis and death. P2X7R is an effective target for the treatment of a range of diseases and has been widely studied in sepsis, neuropsychiatric disorders, acute lung injury, metabolic syndrome, and other diseases. Moreover, studies have also found that targeting P2X7R has a significant effect on the treatment of tumors, nervous system diseases, cardiovascular metabolic diseases, autoimmune diseases, and so on (Ye et al., 2024).

As an ATP derivative, oxidized ATP covalently alters the P2X7R receptor’s ATP binding site, hence permanently impairing its function. It dramatically lowers glial cell activation, neuronal mortality, and the release of inflammatory markers (including IL-1β, TNF-α, and IL-6) in the cerebral ischemia-reperfusion paradigm (Chu et al., 2012).

One possible P2X7R antagonist is A-804598 (N-cyano-N '- [(1S)-1-phenylethyl] -n '-5quinolinyl-guanidine), whose primary pharmacophores are quinoline and cyanoguanidine. Its pharmacokinetic and titer characteristics are excellent. Its inhibitory effect on human P2X7R is strong, but that of mice and rats on P2X7Rs is stronger. A-804598’s pharmacokinetic analysis demonstrated that it could withstand high concentrations in brain tissue, cross the blood-brain barrier, and inhibit P2X7R. A-804598 was recently found to reverse changes in microglia and astrocytes when injected into C57BL/6J mice that had been exposed to ethanol and a high-fat diet for an extended period. It also reduced or eliminated mRNA levels of several inflammatory markers, such as IL-1β, iNOS, and CXCR2, as well as components of the inflammatory signaling pathway, such as TLR2, caspase-1, NF-κB1, and cAMP response element binding protein 1. Furthermore, since no cross-species differences in P2X7Rs were observed, A-804598 could not be quickly translated into preclinical studies. Additionally, A-804598 radiolabeled with [3h] demonstrated strong *in vivo* affinity and selectivity for P2X7R (Lee et al., 2023; Martinez et al., 2015).

AZ-11645373, a thiazolidinedione analogue derived from compound 1, is a highly selective and potent human P2X7R antagonist. It inhibits eATP-mediated IL-1β release *in vitro* (Lee et al., 2023). The P2X7 antagonist AZ11645373 inhibits the expression of inflammatory molecules induced by various classes of agonists, including inflammatory cytokines, bacterial pathogen-associated molecular patterns (LPSs), and endogenous DAMPs (lipid oxidation products such as oxidized 1-palmitoyl-2-arachidonyl-phosphatidylcholine). Besides IL-8, AZ11645373 also inhibits the induction of the prostaglandin-endoperoxide synthase 2 gene, which may be hindered in many ways in the development and transmission of inflammation (Oskolkova et al., 2017).

JNJ-54175446 displayed dose-dependent plasma exposure in study participants without any reported serious adverse effects. In another multiple escalation dose trial spanning from 40 to 450 mg, JNJ-54175446 was found to be well tolerated by participants (Illes et al., 2023). It also shows good blood-brain barrier penetration, with a brain-to-plasma ratio of 1. The compound exhibits high selectivity for P2X7R compared to other P2XRs, including P2X1R, P2X2R, P2X3R, and P2X4R (Lee et al., 2023). In the presence of the P2X7R agonist BzATP, JNJ-54175446 reduced the release of IL-1β in LPS-stimulated peripheral blood cells, attenuated IL-1β/IL-18 release in microglia and attenuated dextroamphetamine-induced (visual) motor performance and sustained attention improvement. At the same time, the effect of dexamphetamine on enhancing subjective mood in healthy volunteers was changed (Recourt et al., 2023).

JNJ-64413739 is selective, metabolically stable, and brain permeable. JNJ-55308942 blocks BZ-ATP-induced brain IL-1β release, it can also reduce the activation of microglia caused by LPS in mice. JNJ-54232334, labelled with 3H radioactive material, was used for binding studies and demonstrated a strong affinity for P2X7R in rats, particularly in humans (Müller et al., 2022).

A-438079 can cross the blood-brain barrier, is a selective P2X7R antagonist, and has been used in a variety of inflammatory disease models with beneficial effects but is considered unsuitable for chronic use due to its short half-life and low bioavailability. It has been shown that inhibition of P2X7R by A-438079 *in vitro* reduces oxLDL accumulation in human podocytes and prevents oxLDL-induced apoptosis (Genetzakis et al., 2022).

Brilliant Blue G (BBG) inhibits K^+^ efflux and Ca^2+^ inflow while preventing the development of P2X7R big pores. Simultaneously, enhance brain ischemia/subarachnoid hemorrhage injury, decrease neuroinflammation, limit astrocyte and microglia activation, and lower IL-1β levels (Ren et al., 2023; Wang et al., 2017; Chen et al., 2013).

A-740003 reduces the release of neurotoxic chemicals including glutamate and ATP by glial cells by inhibiting the P2X7R/NLRP3/IL-1β axis (Ye et al., 2018). The monosodium urate (MSU)-induced maturation of IL-1β was inhibited in the gout model (Gicquel et al., 2015).

ITH15004 is highly selective, more selective to P2X7R than other P2X receptors, halving ATP-induced IL-1β release in LPS-induced macrophage inflammatory response and exhibiting high lipophilic membrane permeability in Parallel Artificial Membrane Permeability Assay, indicating that it can permeate the blood-brain barrier and enter the brain. Higher lipid membrane permeability than other synthetic compounds (Lee et al., 2023; Calzaferri et al., 2021; Rosli et al., 2019).

Based on the aforementioned review, P2X7 receptor agonists and antagonists exhibit considerable potential in preclinical studies. Notably, P2X7 antagonists hold particular promise as therapeutic agents for inflammatory and neurodegenerative diseases. However, further research is essential to optimize their efficacy, enhance their safety profiles, and elucidate their pharmacokinetic properties. Future preclinical studies should prioritize the development of more selective and potent antagonists, conduct comprehensive toxicity assessments, and explore synergistic effects when combined with other therapeutic agents. Moreover, additional investigation into the role of P2X7 agonists in physiological and pathological processes can provide deeper insights into the regulation of the P2X7 receptor and its potential as a therapeutic target.

### 5.2 Pharmacological effects of P2X7R

#### 5.2.1 Subarachnoid hemorrhage

By suppressing the activation of caspase-1 and preventing the assembly of the NLRP3 inflammasome, inhibiting P2X7R can reduce the production of IL-1β/IL-18 and lessen early brain damage in the subarachnoid hemorrhage model. After SAH, Chen S et al. discovered that BBG therapy could reduce brain edema and improve neurobehavioral function while also lowering caspase-1 cleavage and the consequent generation of mature IL-1β/IL-18. BBG’s anti-inflammatory action was finally confirmed when it suppressed neutrophil infiltration in the cortex 24 h after SAH (Chen et al., 2013).

#### 5.2.2 Intracerebral hemorrhage

In the study on the temporal and spatial expression of P2X7R after cerebral hemorrhage and its role and mechanism in protecting the blood-brain barrier disruption, Zhao H et al. found that P2X7R was significantly elevated in the brains of cerebral hemorrhage rats. By injecting the P2X7R inhibitor a-438079 into their bodies, it could partially maintain the integrity of the blood-brain barrier after cerebral hemorrhage by inhibiting RhoA activation and increasing the expression of endothelial tight junction proteins (such as Occludin, VE-Cadherin and ZO-1), thereby reducing neuronal death, neurological dysfunction, blood-brain barrier disruption and brain edema (Zhao et al., 2016).

#### 5.2.3 Cerebral ischemia-reperfusion injury

A recent study has found that inhibiting the activation of P2X7R after cerebral ischemia-reperfusion injury can reduce oxidative stress and inflammation and improve neurological behavioral scores. Chu K et al. performed transient global cerebral ischemia-reperfusion in rats after caudal vein infusion of P2X7R BBG and 5 ′-adenosine triphosphate-2′, 3 ′-dialdehyde (OxATP) for 20 min. The survival rate was calculated. Neuronal death was observed and the expressions of IL-1β, TNF-α, and IL-6 were detected. The results showed that P2X7R inhibition reduced I/R-induced neuronal death and neuronal DNA cleavage while improving neuronal survival. Inhibition of P2X7Rs ameliorates I/R-induced behavioral deficits, and reduces I/R-induced glial activation, and attenuates I/R-induced cytokine overexpression. This suggests that P2X7R antagonists BBG and OxATP reduce the expression of inflammation by reducing the activation of astrocytes and microglia and reducing the release of reduced inflammatory factors (Chu et al., 2012).

#### 5.2.4 Atherosclerosis

In the atherosclerosis model, targeting and inhibiting P2X7R can prevent the formation of foam cells and thereby inhibit lipid accumulation. Genetzakis E et al. discovered that the P2X7R antagonist A-438079 can reduce the expression of ICAM-1 in the cerebral microvascular system, thereby reducing the infiltration of immune cells into the vulnerable areas of the vascular wall. A-438079 can also inhibit the uptake of oxLDL and reduce the rate of macrophage foaming (Aslanian and Charo, 2006; Genetzakis et al., 2022).

#### 5.2.5 Other models

In studies concerning the relationship between P2X7R activation and IL-1β release in satellite glial cells (SGCs) after inflammatory stimulation, Neves AF et al. enriched SGCs with lipopolysaccharide (lps) -treated SGCs. The P2X7R agonist BzATP activated P2X7R to induce IL-1β release in the supernatant, and this effect was blocked by P2X7R antagonist A-740003(237) (Neves et al., 2020). In other studies, the P2X7R antagonist A-74003 was found to reduce the release of glial transmitters such as glutamate, ATP, and D-serine, reducing synaptic activity and plasticity (Ye et al., 2018). To study the dependence of IL-1β production in human macrophages on the activation of the purinergic receptor and the NLRP3 pathway, this study demonstrated the involvement of P2X7R in MSU -induced IL-1β production in macrophages. Treatment of LPS-and MSU-stimulated macrophages with the selective, competitive P2X7R antagonist A-740003 was found to reduce IL-1β release and pro-IL-1β cleavage (Gicquel et al., 2015).

In test P2X7Rs in chronic alcohol and mouse nerve inflammation induced by high-fat diet in the study of the role, Freire D et al. found that chronic alcohol and P2X7Rs expression caused by high-fat diet (mixed) increased, thus increasing nerve inflammation in mice. Treatment of mice with the P2X7R antagonist A804598 reduced inflammatory factors such as IL-1β and abolished mixed-induced neuroinflammation but did not affect the neuroprotective response or alter many neurotransmitter receptors (Freire et al., 2019).

P2X7R has shown its therapeutic potential in cerebrovascular diseases in pharmacological models and is a good therapeutic target. The P2X7R antagonists are still in the preclinical stage, but the results from animal models show significant protective effects and the mechanism is clear. However, the formulation and selectivity still need to be improved.

### 5.3 Translational challenges and future drug development

The current limitations of P2X7R antagonists include insufficient blood-brain barrier BBB penetration. Most P2X7R antagonists (such as A-438079 and BBG) cannot effectively penetrate the BBB due to their molecular weight or hydrophilicity, which limits their efficacy in central nervous system diseases. Although some compounds (such as JNJ-54175446) show certain BBB penetration ability (brain/plasma ratio ≈1), their efficiency still needs to be optimized. There is a significant difference in ATP sensitivity between human and rodent P2X7R, which reduces the predictive value of preclinical data. Although some antagonists (such as A-804598) are effective in animal models, the difference in cross-species activity hinders clinical translation. P2X7R has physiological functions in immune homeostasis, and long-term inhibition may interfere with normal immune surveillance (such as anti-infection ability). Some compounds (such as AZ-11645373) have weak selectivity and may affect other P2XR. Some antagonists are large molecules or charged, making systemic administration difficult. Currently, clinical research progress is slow, and only a few antagonists (such as JNJ-54175446) have entered early clinical trials, lacking large-scale phase III studies to verify their efficacy in cerebrovascular diseases.

The above limitations offer potential directions for development and breakthroughs. The use of high-specificity, small-molecular-weight nanobodies can enhance BBB penetration and reduce off-target risks. By modifying to increase lipophilicity (such as the lipid membrane permeability of IT15004), or by using BBB transporters for active delivery. Developing non-competitive antagonists to avoid completely blocking the physiological function of the receptor. Combining with NLRP3 inhibitors or anti-cytokine drugs to enhance anti-inflammatory effects. Combining with antioxidants (such as edaravone) or autophagy activators (such as rapamycin) for multi-pathway intervention of damage. Utilizing nanocarrier systems for precise drug delivery, such as loading drugs with liposomes or polymer nanoparticles (such as Citrate-Agomelatine NPs) to achieve brain-targeted delivery. Screening patients with P2X7R gene polymorphisms to predict drug responses and conduct precision treatment. Using PET imaging tracers (such as SMW139, [11C] JNJ54173717) to monitor brain P2X7R activity as a biomarker platform for treatment response.

## 6 Discussion

Cerebrovascular disease is a major threat to human life and health, and the prevention and treatment of this disease is of great significance to the protection of patients’ lives and property. The pathogenesis of cerebrovascular diseases has been studied relatively systematically, and these mechanisms include neuroinflammation, autophagy, oxidative stress, iron death, etc. P2X7R, as a non-selective cation channel, can be activated by high concentration of ATP, and it is an important receptor to drive inflammation. P2X7R has been found to play an important role in the progression of many diseases, and intervention in P2X7R activity may help ameliorate these conditions and achieve therapeutic goals. The biological properties of P2X7R indicate that it primarily influences the progression of cerebrovascular diseases by mediating inflammatory responses. Most current studies focus on how P2X7R-mediated neuroinflammatory responses contribute to cerebrovascular diseases, but some also explore mechanisms involving autophagy and oxidative stress. Given P2X7R’s potential therapeutic effects in cerebrovascular disease, researchers have investigated the specific pharmacological actions of P2X7R antagonists and agonists. These studies aim to identify new therapeutic approaches for cerebrovascular diseases, leveraging the receptor’s involvement in inflammatory, autophagic, and oxidative stress pathways.

P2X7R has been extensively studied in CNS diseases such as neurodegenerative diseases and mood disorders, but there has been limited research on P2X7R in the context of cerebrovascular diseases. This may be due to the complex and multifaceted mechanisms underlying cerebrovascular diseases, which have led researchers to focus more on the broader physiopathologic aspects of these conditions. As an important receptor, P2X7R has only recently gained attention in this area. Nonetheless, it is undeniable that P2X7R plays a crucial role in cerebrovascular diseases. Research on this receptor is of great significance for the prevention and treatment of these diseases, potentially leading to novel therapeutic strategies and improved patient outcomes. We have observed that P2X7R plays a complex role in cardiovascular diseases. Activation of P2X7R offers protection against myocardial ischemia/reperfusion injury, yet it can also exacerbate cardiomyopathy by inducing cardiac hypertrophy and fibrosis (Chen et al., 2018). This dual nature suggests that P2X7R may similarly exhibit both protective and adverse effects in cerebrovascular diseases. Specifically, it may provide neuroprotection in the early stages of mild cerebrovascular disease by promoting the release of inflammatory factors. However, in severe cerebrovascular disease, P2X7R activation can lead to Ca^2+^ influx and K^+^ efflux, resulting in intracellular inflammation, oxidative stress, cell death, and ultimately worsening of the disease (Ahn et al., 2023; Domercq et al., 2010).

Existing research has demonstrated that P2X7R plays a crucial regulatory role in the onset and progression of cerebrovascular disease. Given its therapeutic potential, antibodies and nanobodies targeting P2X7R offer promising avenues due to their high specificity and low off-target effects. Nanobodies, in particular, are highly selective for P2X7R and have minimal adverse effects. However, their inability to cross the blood-brain barrier remains a significant challenge.

The application of P2X7R antagonists, monoclonal antibodies, or nanobodies could become promising therapeutic approaches for various cerebrovascular diseases. Emerging research tools such as network pharmacology, histological techniques, single-cell analysis, and brain imaging techniques will be crucial in overcoming current challenges. These tools will facilitate the investigation of neuroprotective roles of herbal medicines and aid in the development of new drugs, particularly for ischemic stroke.

Overall, P2X7R undeniably plays a multifaceted and significant role in cerebrovascular diseases. More researches are needed to fully explore this role. The use of P2X7R antagonists, monoclonal antibodies, nanomaterials, and traditional Chinese medicines related to P2X7R, combined with emerging technologies, holds great potential for discovering new methods of preventing and treating cerebrovascular diseases.

## Glossary

P2X7R: P2X7 Receptor
CAD: coronary artery disease
SAH: subarachnoid hemorrhage
ICH: intracerebral hemorrhage
BzATP: 2′(3′)O-(4-benzoylbenzoyl)-ATP
ATP: adenosine triphosphate
ROS: reactive oxygen species
IL: Interleukin
ERK: extracellular signal-regulated kinase
eATP: extracellular ATP
CNS: central nervous system
HIF-1α: hypoxia-inducible factor 1α
CpG: cytosine-phosphodiester-guanosine
mRNA: mribonucleic acid
Sp1: specific protein 1
DNA: deoxyribonucleotide acids
EBI: early brain injury
DBI: delayed brain injury
MFGE8: milk fat globule epidermal growth factor like 8
DAMPs: damage-associated molecular patterns
TLRs: Toll-like receptors
TNF: tumor necrosis factor
MMP: matrix metalloproteinase
NF-κB: nuclear factor-κB
MAPK: Mitogen-activated protein kinase
STAT3: signal transducer and activator of transcription 3
JAK2: Janus kinase-signal transducer
NOX: nicotinamide adenine dinucleotide phosphate oxidase
iNOS: nitric oxide synthase
BBB: blood-brain barrier
CCL: C-C motif ligand
CCR5: CC chemokine receptor 5
I/R: cerebral ischemia-reperfusion
Akt: protein kinase B
mTOR: mammalian target of rapamycin
OS: oxidative stress
PI3k: phosphatidylinositol 3-kinase
OxLDL: Oxidized low-density lipoprotein
OSE: oxidation specific epitopes
CD: cluster of differentiation
CXCL: CXC chemokine ligand
ADAM: a disintegrin and metalloproteinase
LPS: lipopolysaccharide
JNK: Jun amino-terminal kinases
BBG: brilliant blue G
RhoA: Ras homolog gene family member A
OxATP: 5′-adenosine triphosphate-2′, 3′-dialdehyd
SGCs: satellite glial cells
MSU: monosodium urate
